# Effects of wireless local area network exposure on testicular morphology and VEGF levels

**DOI:** 10.1038/s41598-026-37323-2

**Published:** 2026-01-27

**Authors:** Eser Çakmak, Birşen Bilgici, Begüm Korunur Engiz, Adem Kocaman

**Affiliations:** 1 Medical Biochemistry Department, Faculty of Medicine, Ondokuz Mayıs University, Samsun, 55139 Turkey; 2 Electrical and Electronics Engineering Department, Faculty of Engineering, Ondokuz Mayıs University, Samsun, 55139 Turkey; 3 Histology and Embryology Department, Faculty of Medicine, Ondokuz Mayıs University, Samsun, 55139 Turkey; 4 Department of Medical Biochemistry, Ondokuz Mayıs University Faculty of Medicine, Samsun, 55139 Turkey

**Keywords:** Electromagnetic field, VEGFA, HIF1A, Testis, Biochemistry, Biophysics, Health care

## Abstract

This study investigated the effects of exposure to a 2.45 GHz electromagnetic field (EMF) on rat testicular tissue, focusing on histological alterations and the potential activation of the HIF1A-VEGF pathway. Twenty-four adult male albino Wistar rats were divided into a control group (*n* = 12) with no EMF exposure and an experimental group (*n* = 12) exposed to 2.45 GHz microwave radiation (3 V/m, SAR 0.00208 W/kg) for one hour daily over 60 days. Following the exposure period, testicular tissues were analyzed for histopathological changes, VEGFA and HIF1A gene expression levels, and VEGFA protein concentration. In rat testicular tissue, while VEGFA gene expression (*p* < 0.05) and protein levels (*p* < 0.001) increased in the EMF group, no significant change was detected in HIF1A gene expression levels in the EMF group compared to the control group. Histological examination revealed a significant reduction in seminiferous tubule diameter (*p* < 0.001), epithelial thickness (*p* < 0.001), tubule density (*p* < 0.001), and Sertoli cell count (*p* = 0.0098) in the EMF-exposed group. It may be concluded that EMF at 2.45 GHz increases HIF1A-independent VEGF levels, and EMF exposure may cause testicular damage by increasing VEGF gene expression levels.

## Introduction

In daily life, individuals are routinely exposed to electromagnetic fields (EMF) emitted by sources such as power lines, computers, televisions, radios, telephones, and Wireless Fidelity (Wi-Fi), often without their explicit awareness. Scientifically, EMF is defined as the space in which moving electrically charged particles remain under the influence of a force^1^. The biological effects of EMF exposure can vary significantly among individuals. Factors such as gender, body tissue density, and exposure duration influence this variability, as do physiological characteristics including body weight, body mass index, bone density, and water-electrolyte balance. These factors can modulate the body’s conductivity and biological reactivity to EMF, leading to divergent physiological responses^2,3^. At the cellular level, EMF exposure can trigger a spectrum of biological and chemical reactions. These may include alterations in cell proliferation^4,5^, changes in cell morphology and plasma membrane structure, modifications in gene expression^6,7^, and the induction of apoptosis^8^. Furthermore, EMF has been associated with more severe outcomes such as DNA damage and mutations, chromosomal abnormalities^9^, hematological disorders^10^, congenital disabilities^11^, inflammatory processes^12^, and the development of various cancers^13^. The effects of EMF on the reproductive and genital systems have become a subject of research today^14^. It has been reported that exposure to EMF reduces antioxidant enzyme activities in the testes by causing oxidative stress^15–17^, has adverse effects on the human reproductive system by disrupting the structure and motility of spermatozoa^18,19^, may cause structural deterioration in seminiferous tubules^16,20^, induces apoptosis in testicular germ cells^21^, leads to DNA damage in the testes^22^, and may negatively affect fertility by causing functional and structural changes in the male reproductive system^15,23,24^.

VEGF is the most important regulator of endothelial growth and permeability in the male reproductive system^25^. It is synthesized in the male genital tract from Sertoli and Leydig cells, as well as from prostate epithelial cells, peritubular cells of the epididymis, and seminal vesicles. VEGF is crucial for germ cell homeostasis and can regulate male fertility in the testes^25–27^. The VEGF family consists of five different ligands: VEGF (VEGFA), VEGFB, VEGFC, VEGFD, and Placental Growth Factor. The best-characterized member of this family is VEGFA, a 45 kDa dimeric N-glycoprotein^28^. It plays a role in many physiological and pathological processes, including hematopoiesis, angiogenesis, endocrine function, inflammation, neurogenesis, vascular remodeling, vasculogenesis, metabolism, vascular survival, and vascular permeability^29^. The effects of VEGF occur through Vascular Endothelial Growth Factor Receptor 1 (VEGFR1) and Vascular Endothelial Growth Factor Receptor 2 (VEGFR2). The primary receptor through which VEGF acts in the neovascularization process is VEGFR2, which has a tyrosine kinase signaling domain^30^.

VEGF expression is regulated by diverse stimuli, including hypoxia, oncogenes, cyclooxygenase-2 (COX2), and nitric oxide. Its induction is further mediated by various growth factors—such as Insulin-like Growth Factor-1 (IGF-1), Epidermal Growth Factor (EGF), Basic Fibroblast Growth Factor (bFGF), Platelet-Derived Growth Factor (PDGF), and Transforming Growth Factor-β (TGF-β)—as well as by proinflammatory cytokines, including Interleukin-1β (IL-1β), Interleukin-6 (IL-6), Interleukin-8 (IL-8), and Tumor Necrosis Factor-α (TNF-α)^31^. Hypoxia, one of the pathways that increases VEGF expression, leads to the activation of transcription factors such as Hypoxia Inducible Factor 1 Subunit Alpha (HIF1A)^32^nder normoxic conditions, the ubiquitination and proteasomal degradation of HIF1A prevent its dimerization and binding to VEGF promoter regions, thereby suppressing VEGF expression. On the other hand, under hypoxic conditions, HIF1A is not ubiquitinated and dimerizes, stimulating VEGF expression and angiogenesis^33^.Another molecular pathway is that COX upregulates VEGF expression via Prostaglandin E2 (PGE2)^34^.

While acute inflammation is a response of cellular defense, chronic inflammation can lead to a wide range of diseases, including cancer. Inflammation and tumorigenesis processes are intertwined and quite complex. VEGF, which plays a key role in these processes, is reported to be critical in suppressing apoptosis, inducing proliferation, and promoting angiogenesis, invasion, and metastasis^35^.

The scientific literature contains studies linking EMF exposure to pathological conditions such as inflammation^12,36^, infertility^23^, and cancer^13^. However, findings from research on the effects of EMF on testicular tissue are inconsistent and controversial. VEGF, which plays a role in many physiological and pathological processes, is the primary regulator of endothelial growth and permeability in the male reproductive system. Currently, there is no study in the literature investigating the relationship between EMF exposure and the levels of VEGF and HIF1A in testicular tissue. Therefore, this study aimed to examine whether VEGF—a factor widely expressed in testicular tissues—is affected by exposure to 2.45 GHz microwave-frequency EMF, as emitted by Wi-Fi devices common in daily life. A further objective was to determine whether VEGF expression in rat testicular tissue is stimulated via the HIF1A molecular pathway and to examine concomitant morphological changes in the testis.

## Materials and methods

### Animal experimentation

This study was conducted with the approval of the Ondokuz Mayıs University Animal Experiments Local Ethics Committee (2020/36). Animals were handled with all precautions necessary to avoid suffering in accordance with the ARRIVE guidelines. All experiments performed were following relevant guidelines and regulations. For this purpose, a total of 24 adult male albino Wistar rats (obtained from Ondokuz Mayıs University Experimental Animal Research Center), each weighing between 200 and 300 g, were utilized. The rats were housed in an environment where they had ad libitum access to commercially available rat pellets, and distilled water was provided as drinking water. The lighting in the experimental environment was programmed to follow a 12-hour light and 12-hour dark cycle, with the temperature maintained at 22 ± 1 °C. In our study, to minimize restraint stress, a standard habituation protocol was applied to all animals. Five days prior to the experiment, all rats were placed in a restrainer for one hour daily, without EMF exposure, to allow for acclimatization and stress reduction. The animals were randomly assigned to either the control (*n* = 12) or EMF-exposed (*n* = 12) group. Of all 24 rats, while the control group (*n* = 12) was not exposed to EMF but placed in a restrainer, the EMF group (*n* = 12) was exposed to 2.45 GHz MW radiation for 1 h a day for 60 days in a 12-slice pie cage restrainer.

### Application of the electromagnetic field

In this study, a monopole antenna Microwave system (2004X-RF, Everest Co., Adapazarı, Turkey) was used to generate the 2.45 GHz frequency generated by WLAN systems. The microwave system is capable of emitting continuous or discrete waves at 2.45 GHz, with 0–1 W of output power and 217 Hz modulation. At the beginning of the study, before the 2.45 GHz MW system was started, the Narda SRM-3006 EMF meter was used to measure possible reflections and electric fields in the environment, and the services and their contributions that constitute the noise floor were determined.

MW exposure was applied in a 12-slice pie cage restrainer and the antenna was placed at the midpoint of both pie cage restrainers to ensure equal distribution of the electric field. Control group rats and rats exposed to EMF were kept in a separate room free of EMF pollution after MW exposure. During the experiment, temperature and EMF measurements were taken from 3 rats in the control and study groups once a week. During MW exposure, electric field measurements were made on three rats, from the head, body and tail parts for 6 min according to the Information Technologies Authority and ICNIRP regulations. With the help of the electric field values, the specific absorption rate (SAR) was calculated for each rat via the CST program, and the maximum SAR (10 g) was measured as 0.00208 W/kg.

At the end of 60 days, each rat was anesthetized with 100 mg/kg ketamine hydrochloride intraperitoneally, and all rats sacrificed by cervical dislocation. Testis tissues were removed for measurement of gene expression and protein levels and stored at −80 °C until the study day. For the histopathological examination, after reperfusion with saline, the testis tissues were dissected and placed in 10% formalin. To prevent potential bias, tissue samples collected from the animals were anonymized by a laboratory worker responsible for animal care, not involved in the study, using a random coding system. All laboratory procedures were performed using these codes without any indication of group identity. The blinding was lifted only after all analyses were completed.

### Biochemical analyses

#### Measurement of gene expression levels by quantitative polymerase chain reaction (qPCR)

Testis samples were homogenized with liquid nitrogen. Using TRIzol (One Step RNA Reagent, Bio Basic UN2922), RNA isolation was performed. The quality and quantity of RNAs were evaluated using a Nanodrop spectrophotometer. Using iScript cDNA Synthesis Kit (BIORAD, Cat. No: 1708891) mRNAs were converted to cDNA. Using iTaq Universal SYBR Green Supermix (BIORAD, Cat. No: 1725121) The GAPDH, VEGFA and HIF1A genes expression were measured via qPCR. The primer sequences of the genes are given in Table 1.

**Table 1:** Sequences of polymerase chain reaction primers.

Gene	Forward primer	Reverse primer
GAPDH	GCAAGTTCAACGGCACAGTCAAG	GCCAGTAGACTCCACGACA
VEGFA	AGAAAGATAGAACAAAGCCAGAAAA	TCTAGTTCCCGAAACCCTGA
HIF1A	AAGCACTAGACAAAGCTCACCTG	TTGACCATATCGCTGTCCAC

#### Measurement of protein levels by ELISA

The tissue homogenate was added to 1 mL of phosphate-buffered saline (PBS; pH 7.4) and vortexed. Subsequently, the samples were centrifuged at 3000 × g for 20 min, and the supernatants were collected for ELISA analysis. Protein concentrations were determined using the Lowry assay^37^. VEGF levels were quantified using a commercial ELISA kit (Cat. No: 201-11-0660, SunRed). For the rat VEGF ELISA kit, the inter-assay CV was < 11% and the intra-assay CV was < 9%. Kit sensitivity was 10.127 ng/L. The measurement range was between 11 and 3000 ng/L. Absorbances were measured at 450 nm wavelength in an ELISA reader (BIOTEK SYNERGYTM 4, Serial No: 233513, USA) and the results of testicular tissue VEGF levels were expressed as pg/mg protein.

### Histological analyses

The diameters of 20 randomly selected seminiferous tubules from tissue sections of each animal were measured. Tubules exhibiting the most circular morphology and well-defined borders were preferentially selected. Measurements were conducted using ImageJ software (NIH, USA). The obtained data were analyzed to assess whether there was any narrowing of tubule diameters due to the influence of EMF between the groups. To evaluate spermatogenetic activity and the presence of atrophic changes, the thickness of the tubule epithelium was determined by measuring the distance between the basal membrane and the lumen’s starting point in each seminiferous tubule. At least four measurements were taken from different points within each tubule to obtain average values. To gain insight into seminiferous tubule density, tissue organization, and the extent of degeneration, the number of seminiferous tubules within a 1 mm² area was quantified in randomly selected regions of testis sections. To assess potential changes in the Sertoli cell population due to EMF exposure, Sertoli cells were manually counted in 20 selected seminiferous tubules from testis sections.

### Statistical analysis

Relative expression software (REST) QIAGEN 2009 analysis used for relative gene expression. GAPDH was accepted as a reference gene, and the control group was accepted as calibrator.

SPSS 22.0 (Statistical Package for Social Sciences) software was used for the statistical evaluation of VEGF protein levels in testicular tissue. The normal distribution of the data was tested using the Shapiro-Wilk normality test. Since the data conformed to a normal distribution, the Student t-test was used to compare parametric data.

For histological examination, data of morphological parameters are presented as mean ± standard deviation (SD). Statistical comparisons between groups were performed using appropriate tests based on the distribution of the data. For normally distributed data, an independent-samples t-test was used. Data analysis was conducted using GraphPad Prism 8 (Prism 8.0) software.

## RESULTS

### Gene expression levels

**Fig. 1:**
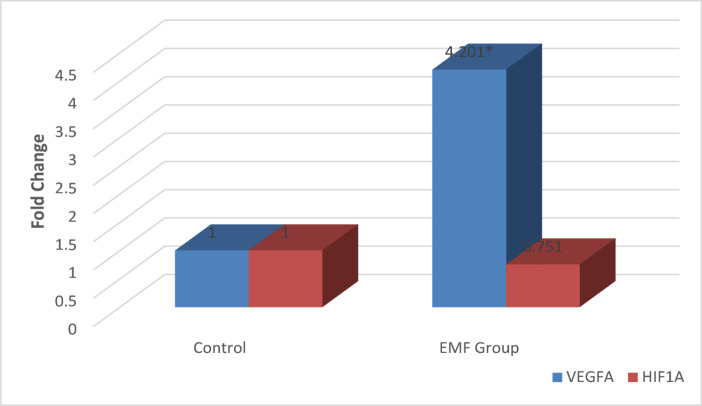
Gene expression changes of VEGFA and HIF1A in rats.* *p* < 0.05.

In rat testis tissues, VEGFA gene expression levels increased by 4.201-fold in the EMF group (*p* < 0.001), whereas HIF1A gene expression levels did not differ significantly between the EMF and control groups (*p* = 0.240) (Fig. 1).

### Protein levels

VEGFA protein levels in rat testicular tissues increased in the EMF group compared to the control group (*p* < 0.001) (Table 2).

**Table 2:** Comparison of VEGFA protein levels between EMF and control groups.

	Control group (*n* = 12)	EMF group (*n* = 12)	*p*-value
VEGFA protein levels (pg/mg protein)	50.42 ± 12.30	81.49 ± 11.78	*p* < 0.001

Data are presented as mean ± standard deviation (SD).

### Histological examination

**Fig. 2:**
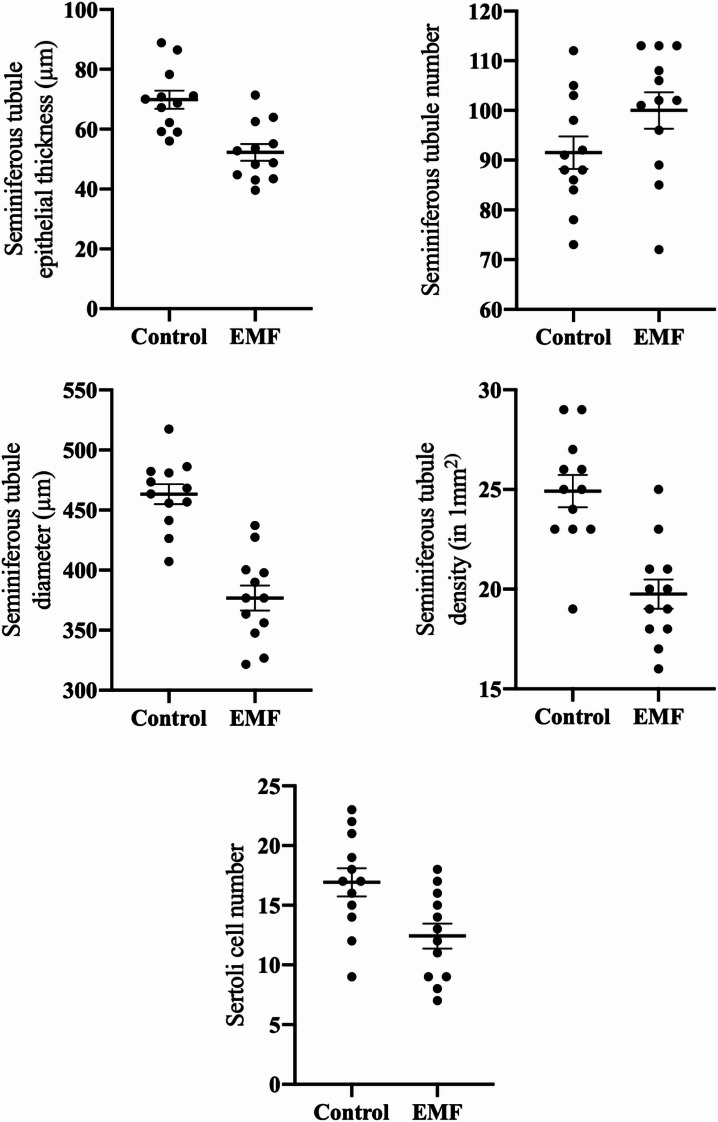
Statistical analysis of morphometric changes in testicular tissue associated with EMF exposure.

**Fig. 3:**
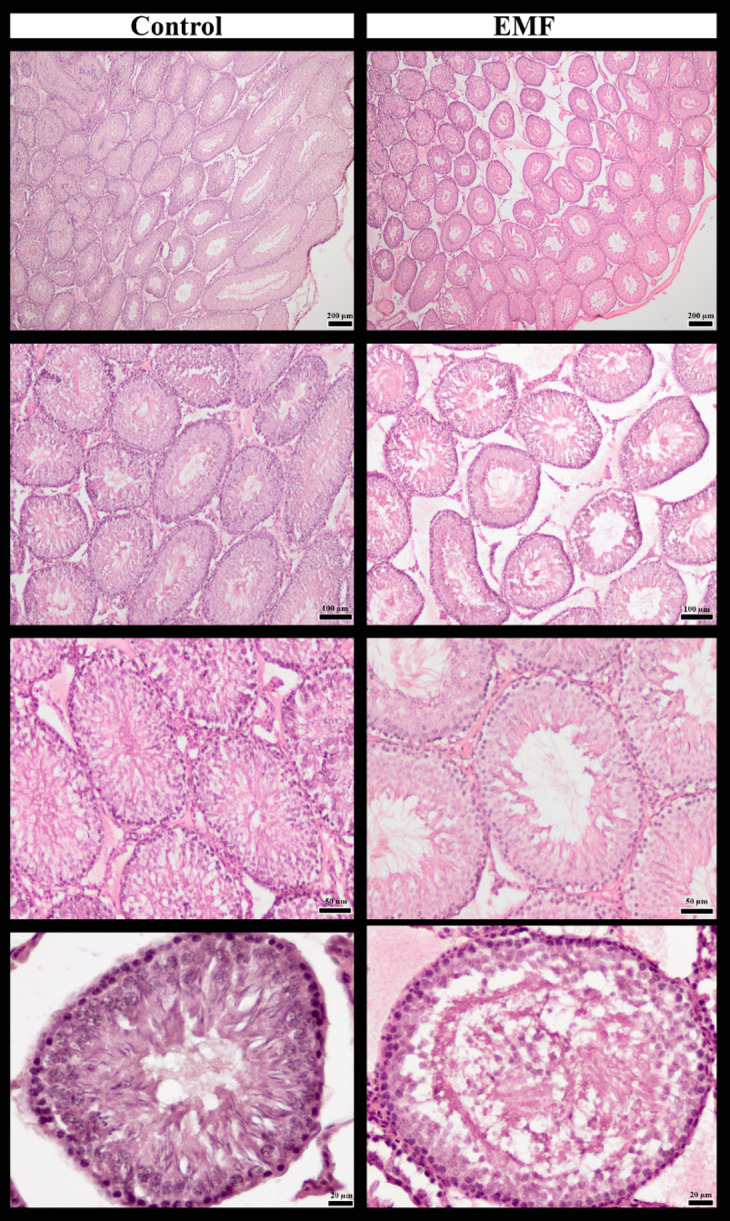
Histological appearance of seminiferous tubules (200 μm, 100 μm, 50 μm, 20 μm) in the testes of control and EMF groups (H&E staining).

The seminiferous tubule diameter was significantly smaller in the EMF group compared to the control group (*p* < 0.001). The number of seminiferous tubules showed no significant change between the EMF and control groups (*p* = 0.0971). The thickness of the tubule epithelium was significantly reduced in the EMF group compared to the control group (*p* < 0.001). Seminiferous tubule density was significantly decreased in the EMF group compared to the control group (*p* < 0.001). Finally, the number of Sertoli cells was also significantly reduced in the EMF-exposed group compared to the control group (*p* = 0.0098) (Table 3; Figs. 2 and 3).

**Table 3:** Comparison of testicular morphometric parameters between EMF and control groups.

	Control group (*n* = 12)	EMF group (*n* = 12)	*p*-value
Seminiferous Tubule Diameter (µm)	463.2 ± 13.41	376.8 ± 13.41	< 0.001
Seminiferous Tubule Count	91.50 ± 4.90	100.0 ± 4.90	0.0971
Tubule Epithelial Thickness (µm)	69.84 ± 4.10	52.28 ± 4.10	< 0.001
Tubule Density (tubules/mm²)	24.92 ± 1.09	19.75 ± 1.09	< 0.001
Sertoli Cell Count	16.92 ± 1.59	12.42 ± 1.59	0.0098

Data are presented as mean ± standard deviation (SD).

## Discussion

Wi-Fi technology has become an inseparable part of our lives and emits EMF, and the controversial effects on human health, especially on testicular tissue, have prompted studies in this area. In this study, the effects of EMF at 2.45 GHz frequency (3 V/m, SAR (10 g) 0.00208 W/kg) on the gene expression levels of VEGFA and HIF1A proteins in rat testicular tissue were investigated and a statistically significant increase was found in VEGFA gene expression and protein levels in the EMF group compared to the control group, while no statistically significant difference was found in HIF1A gene expression levels. Histological examination revealed a significant reduction in seminiferous tubule diameter, epithelial thickness, tubule density, and Sertoli cell count in the EMF-exposed group.

The testes are very sensitive to electromagnetic radiation, and exposure to EMF can cause testicular damage^38^. A study showing that exposure to 2.4 GHz Wi-Fi in rats reduced sperm function indicated that there should be great concern about exposure to Wi-Fi networks near our living spaces^39^. On the contrary, it was reported that rats exposed to 1.95 GHz EMF for 5 h/day for 5 weeks did not show any changes in tissue or reproductive system parameters, and that sperm counts increased^14^. It has been shown that EMFs cause cell death in testicular germ cells in mice^21^, and at high frequencies, especially 2.45 GHz, EMFs reduce Leydig cell numbers and increase apoptosis in seminiferous tubules in rats^40^. In a study examining the effect of EMF at 2.45 GHz on testicular tissue, it was reported that EMF caused DNA base changes and may have mutagenic potential^22^. Exposure to 900 MHz–1.7 GHz has been reported to cause DNA breaks in embryonic stem cells and epididymal spermatozoa of mice^9^. It has been suggested that testicular germ cell apoptosis can be induced even with extremely low-frequency magnetic field exposure^21^, and this frequency directly causes DNA strand breakage in testicular cells in mice^41^.

Articles examining the effect of EMF on VEGFA and HIF1A are limited in the literature, and the results vary depending on the tissue/cell type examined and the intensity and duration of EMF. In a study using a mouse model of myocardial infarction, exposure to a pulsed electromagnetic field (PEMF) at 15 Hz (1.5 mT) and 30 Hz (3.0 mT) increased VEGF and HIF1A mRNA levels in heart tissue^42^. It has been reported that combined exposure of rats to PEMF (27.12 MHz, 12 times a day at 30-minute intervals) and radiofrequency electromagnetic field (0.5 mT, continuous) increases VEGF expression^43^. In a study conducted with 1321N1 cells, it was reported that PEMF exposure induced VEGF release independently of HIF1A, findings consistent with our study despite differences in tissue type, EMF frequency, and exposure duration^44^. In our study, no change in HIF1A gene expression was observed in the EMF group, whereas significant increases in VEGFA gene expression and protein levels were detected, suggesting that VEGF induction occurs independently of HIF1A. According to our results, EMF might have induced VEGF gene expression through activation of the PGE2 pathway of COX^34^, the TGF-β/SMAD signaling pathway^45^, the effects of growth factors^46^, or the NFKB signaling pathway^35^, independently of the HIF1A pathway.

Can EMF exposure cause testicular damage by increasing VEGF levels? The literature reports that 900 MHz EMF can cause inflammation^12^, and we previously showed that EMF at 2.45 GHz increases inflammation and testicular damage^36^. The increase in VEGF levels observed in the EMF group in our study may indicate testicular damage due to inflammation.

It has been proposed that exposure to EMF induces significant histopathological and morphological alterations in the testicular parenchyma, with the seminiferous tubules as the primary target. Evidence from rodent models indicates a marked reduction in seminiferous tubule diameter, findings which are directly correlated with EMF exposure at various frequencies, including 800 MHz and 2.45 GHz^47–49^. These structural deformities, which include irregularly shaped tubules and epithelial cell abnormalities, collectively disrupt the spermatogenic microenvironment and functional testicular integrity^50,51^. Given their critical role in providing structural and metabolic support to germ cells and in maintaining the integrity of the blood-testis barrier, the loss of Sertoli cells severely disrupts the spermatogenic cycle and the delicate tubular microenvironment^52,53^. Further compromising the spermatogenic microenvironment is a significant decrease in tubule epithelial thickness, reflecting a deterioration in the structural organization and proliferative capacity of the germinal epithelium^40,54^). Our findings of reduced seminiferous tubule diameter, epithelial thickness, tubule density, and Sertoli cell count in the EMF-exposed group are consistent with this body of evidence, thereby supporting the notion that EMF can induce testicular damage.

## Conclusions

Depending on the administered dose and duration, EMF may disrupt the testicular microenvironment through mechanisms such as oxidative damage and inflammation. This disruption can lead to an upregulation of VEGF via an HIF1A-independent pathway, such as activation of the PGE2 pathway of COX, the TGF-β/SMAD signaling pathway, the effects of growth factors, and the NFKB signaling pathway, ultimately resulting in testicular damage. Such a pathophysiological progression could consequently lead to dysfunction of the male reproductive system. Given the variations in exposure duration and frequency, the effects of both acute and chronic exposure to EMF at levels commonly encountered in daily life warrant further investigation in future studies.

## Data Availability

All data generated or analyzed during this study are included in this published article.
